# Clinical outcomes of vinorelbine loading CalliSpheres beads in the treatment of previously treated advanced lung cancer with progressive refractory obstructive atelectasis

**DOI:** 10.3389/fbioe.2022.1088274

**Published:** 2022-12-20

**Authors:** Xu Ma, Di Zheng, Jie Zhang, Yu Dong, Lingling Li, Bing Jie, Sen Jiang

**Affiliations:** ^1^ Department of Radiology, Shanghai Pulmonary Hospital, School of Medicine, Tongji University, Shanghai, China; ^2^ Department of Oncology, Shanghai Pulmonary Hospital, School of Medicine, Tongji University, Shanghai, China

**Keywords:** drug-eluting beads, bronchial arterial chemoembolization, anchoring effect, advanced lung cancer, atelectasis

## Abstract

**Background:** Drug-eluting beads bronchial arterial chemoembolization (DEB-BACE) has been used in the treatment of locally advanced lung cancer and has the potential to improve outcomes and reduce recurrence. However, DEB-BACE shows a poor therapeutic effect in advanced lung cancer after failure of multiple therapies. This study assessed the effect of DEB-BACE in the treatment of progressive lung cancer with refractory obstructive atelectasis.

**Methods:** Progressive advanced lung cancer patients with refractory obstructive atelectasis were voluntarily enrolled in this study after failure of multiple conventional therapies. Baseline information, DEB-BACE treatment process, and changes in clinical symptoms were recorded. The primary endpoints were the objective response rate (ORR) and improvement rate of dyspnea. The secondary endpoints were time-to-progression (TTP), overall survival (OS), and rate of pulmonary re-expansion. Treatment-related adverse events and serious adverse events were analyzed to assess the safety of DEB-BACE. The Cox regression model was performed to analyze the possible factors impacting prognosis of DEB-BACE.

**Results:** DEB-BACE was successfully performed with CalliSpheres beads loaded with vinorelbine in the 20 enrolled patients. ORR and disease control rate were 80% and 85%, respectively, at the first follow-up (43.4 ± 15.26 days). The improvement rate of dyspnea was 85% and 80% at 1 week and 1 month (*p* < 0.0001, *p* < 0.0001), respectively. TTP was 41.25 ± 14.43 days and 89.55 ± 61.7 days before and after DEB-BACE, respectively; DEB-BACE delayed the progression of advanced lung cancer (*p* < 0.0001). OS was 238.03 ± 33.74 days (95% confidence interval: 171.9–304.16). The rate of pulmonary re-expansion was 80% at the first follow-up. The reasons for poor prognosis were tumor necrosis, longer disease duration, and pulmonary atelectasis duration (*p* = 0.012, *p* = 0.038, *p* = 0.029). Massive hemoptysis was observed in two cases, and one patient died of asphyxia caused by hemoptysis. Moderate hemoptysis occurred in one case. All three adverse events were considered as the result of the tumor cavity after DEB-BACE.

**Conclusion:** DEB-BACE loaded with vinorelbine is a feasible option for progressive advanced lung cancer with obstructive atelectasis after failure of other treatments.

## Introduction

The incidence of advanced lung cancer has increased in recent years, and lung cancer has become the primary cause of cancer-related death in China (Allemani et al., 2018; Ward et al., 2020). Despite an increasing number of therapies available in clinical practice, the 5-year overall survival rate of advanced lung cancer patients has not significantly improved (Ward et al., 2020). Drug resistance and therapy intolerance are inevitable in advanced stage lung cancer patients (Du and Morgensztern, 2015; Xie and Zhang, 2016; Shaw et al., 2019; Di Federico et al., 2022; Le et al., 2022; Lu et al., 2022). Furthermore, adverse events (AEs) in response to treatments have a serious impact on patient health and therapy response. Among the reported AEs, obstructive pneumonia and atelectasis are key effect-related factors, and progressive obstructive pneumonia and atelectasis can lead to termination of treatment (Ou et al., 2008; Marchioni et al., 2020).

Bronchial arterial infusion chemotherapy (BAI) and bronchial arterial chemoembolization (BACE) are effective therapies for advanced lung cancer, and some studies have shown that these treatments have better efficacy with milder adverse reactions than conventional chemotherapy (Nakanishi et al., 2012; Yuan et al., 2013; Alharbi et al., 2019; Men et al., 2022). The bronchial artery (BA) is the nutrient vessel of tumor mass, and infusion of chemotherapy through the BA and embolization of BA can lead to inhibition of tumor growth (Eldridge et al., 2016; Yu and Hu, 2020). Drug-eluting beads bronchial arterial chemoembolization (DEB-BACE) is a novel slow-releasing drug carrier and embolization system that shows improved efficacy over BACE (Lewis and Holden, 2011; Lewis and Dreher, 2012; Choi et al., 2014). Many studies have reported the efficacy of DEB-BACE on newly diagnosed non-small cell lung cancer (NSCLC), but large tumors and tumors that received previous treatments still showed a poor prognosis after DEB-BACE in some reports (Bie et al, 2019; Li et al., 2021; Liu J et al., 2021; Xu et al., 2022). Therefore, the efficacy of DEB-BACE in the treatment of previously treated NSCLC remains unclear.

The aim of our study was to evaluate the efficacy of DEB-BACE on progressive, previously treated, advanced lung cancer.

## Materials and methods

### Patients

This prospective single-arm clinical study was approved by the local ethics committee, and informed consent was obtained from all patients before DEB-BACE. This study included patients with stage IIIB, IIIC, and IV lung cancer with obstructive atelectasis after failure of conventional therapies. The other inclusion criteria were as follows: 1) blood cell count and blood biochemistry indexes met the criteria for chemotherapy, 2) age between 18 and 85 years old, and 3) voluntary participation in the study and compliance with the follow-up plan. The major exclusion criteria were as follows: 1) researchers determined that the subject was not suitable for DEB-BACE in clinical practice, 2) absence of a valid nutrient vessel for the tumor mass on computed tomography angiography (CTA), 3) existence of serious systemic arterial pulmonary circulation shunts (SPS), and 4) allergic to selected chemotherapy drug and iodine contrast medium. All patients were followed up until death or the last attendance (September 2022).

### Therapeutic process

Vinorelbine was selected as the loading drug following previous studies and the oncologist’s suggestion (Table 1). CalliSpheres beads (Jiangsu Hengrui Medicine Co., Ltd., Jiangsu, China) with a diameter of 300–500 µm were selected as the embolic material and drug carrier. DEB-BACE was performed by three radiologists with over 15 years of experience following the same treatment protocol.

**TABLE 1:** Loadable chemotherapy drugs and dosage recommendations.

Loading drug	Recommended concentration (dosage)	Loading period			References
		5 min (mg)	15 min (mg)	30 min (mg)	
Doxorubicin	20 mg/ml (80 mg)	70	71	72	Zhang et al. (2017)
Irinotecan	20 mg/ml (100 mg)	74	78	80	Chen. Q et al. (2020)
Gemcitabine	20 mg/ml (400 mg)	84	85	92	Bie, Li, Li, Wang, Li, et al. (2019)
Oxaliplatin	5 mg/ml (80 mg)	18	20	20	Han et al. (2019)
Arsenic trioxide	10 mg/ml (60 mg)	5	10	15	Duan et al. (2020)
Vinorelbine	10 mg/ml (40 mg)	19	36	36	Wang et al. (2020)
Raltitrexed	2 mg/ml (4 mg)	1.1	1.7	2.1	Wang et al. (2020)

**FIGURE 1:**
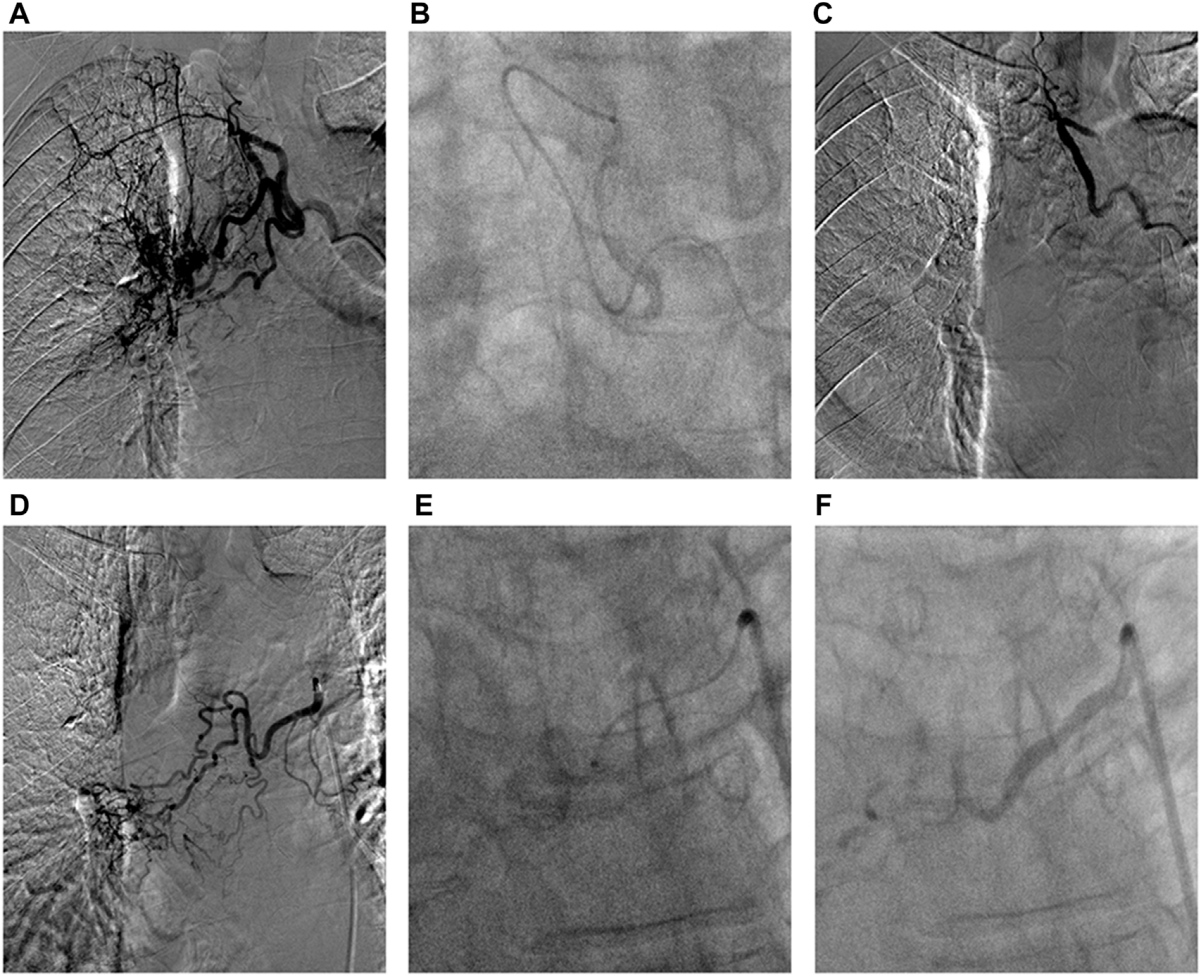
The DEB-BACE procedure in advanced squamous cell carcinoma of the right lung. **(A)** Arteriography showed the intercostal bronchial artery was one main feeding artery of tumor mass; **(B)** The super-selective catheterization with a coaxial microcatheter system (1.98F) was performed to avoid the intercostal artery, and then infuse vinorelbine loaded beads; **(C)** There was no obvious tumor staining after successful embolization; **(D)** One right bronchial artery was considered as the other feeding artery of tumor; **(E)** The super-selective catheterization was performed to infuse vinorelbine loaded beads; **(F)** During second angiography, the distal arterial branches of the right bronchial artery was not observed after successful DEB-BACE.

First, chest CTA scan (slice thickness, 0.625 mm, slice gap, 0.625 mm) was performed to assess the relevant feeding arteries. Second, angiography was performed through various types of 4F or 5F catheters following the CTA through the femoral artery access to confirm the feeding arteries. Tumor staining and degree of SPS were evaluated by three experienced radiologists following methods of previous studies (Nakanishi et al., 2008; Nakanishi et al., 2012; Takeuchi et al., 2020; C. J. Zhang et al., 2022). Next, all confirmed relevant arteries underwent super-selective catheterization with a coaxial microcatheter system that used 4F or 5F outer catheters. Finally, the DEB system, which consists of 1 g CalliSpheres beads loaded with 10 mg vinorelbine, was mixed with iodine contrast medium to 10 ml. The drug-loaded beads were slowly infused (1 ml/min) using a microcatheter system until microvascular casting under fluoroscopy. Successful embolization was defined as no obvious tumor staining during second angiography (Figure 1).

### Evaluation and follow-up

All patients were followed up 1 week after DEB-BACE; blood test results and clinical symptoms were recorded. CTA was recommended once a month to assess tumor response. Tumor response was evaluated using Response Evaluation Criteria in Solid Tumors (RECIST) version 1.1. The ORR was defined as the ratio of patients who achieved complete response (CR) and partial response (PR) among all participants. DCR was defined as the ratio of patients who achieved CR, PR, and stable disease among all participants. The time-to-progression (TTP) was defined as the period from DEB-BACE to progressive disease (PD) or death. The overall survival (OS) was defined as the period from DEB-BACE to death or last follow-up. CTA, blood test results, and clinical symptom changes were recorded each month until PD. After PD of the targeted tumor mass, patients were followed up every 2 months to record treatment and survival.

The primary endpoints of this study were ORR and improvement rate of dyspnea. The secondary endpoints were TTP, OS, and rate of pulmonary re-expansion. The treatment-related AEs included ectopic embolism (e.g., cerebral infarction and pulmonary embolism), chemotherapy-related adverse reactions (e.g., myelosuppression, anorexia, nausea, and liver function impairment), and postembolization syndrome (e.g., hemoptysis, vomit, fever, and chest pain). To assess the safety of DEB-BACE, all AEs were recorded.

### Statistical analysis

The results are expressed as mean ± standard deviation (SD) or number or frequency (percentage). Statistical analysis was performed using SPSS version 22.0 (IBM Corp., Armonk, NY, United States). The rank sum test was applied in the comparation of symptom changes before and after DEB-BACE. The Cox regression model was used to assess the potential risk factors that could affect therapeutic effect. The difference of TTP before and after DEB-BACE was analyzed with Kaplan–Meier curves and the log-rank test. OS was described with Kaplan–Meier curves. A *p*-value < 0.05 was considered statistically significant.

## Results

### Patient characteristics

We identified 23 patients with stage IIIB, IIIC, and IV lung cancer with obstructive atelectasis after failure of conventional therapies between May 2021 and June 2022 at our institution. One patient failed to receive DEB-BACE because of the COVID-19 pandemic and two patients refused to follow up after one DEB-BACE. A total of 20 patients were ultimately enrolled in this study, and 15 patients were diagnosed with squamous cell carcinoma. The average disease duration was 513.86 ± 408.57 days (range: 81–1670 days). The mean duration of pulmonary obstructive atelectasis or pneumonia was 87.5 ± 17.2 days. All enrolled patients previously received intravenous chemotherapy (IVCT), and 14 patients had been treated with two different types of chemotherapy. Ten patients had received radiotherapy (RT). Ten patients had also been treated with immunotherapy targeting programmed cell death 1 (PD-1). Three patients were previously treated with molecular targeted therapy (MTT). Approximately 60% of the patients (12/20) were already intolerant of current treatments (ECOG score: 3/10, 4/1). The baseline clinical features of all subjects are listed in Table 2.

**TABLE 2:** Baseline characteristics of the enrolled patients.

Parameters	Value
Age [M ± SD (range)]	63.55 ± 7.86 (48–77)
Sex (male/female)	19/1
ECOG status (1/2/3/4)	1/8/10/1
Pathology (*n*/%)	
Adenocarcinoma	4 (20%)
Squamous cell carcinoma	15 (75%)
SCLC	1 (5%)
TNM Stage (IIIB/IIIC/IVA)	5/4/11
Disease duration [M±SD (range)]	513.86 ± 408.57 (81–1670)
Time of pulmonary obstructive atelectasis or pnesmonia (M±SD (range))	87.5 ± 17.2
Tumor stage (solid/necrosis)	10/10
Obstruction position (main/lobar bronchus)	7/13
Received treatments (1/2/3/4)	2/8/8/2
Degree of obstruction (pneumonia/atelectasis)	6/14

### Treatment status

DEB-BACE was successfully performed in all 20 subjects. Among the 20 patients, 1 patient showed low grade tumor staining, 7 patients showed medium grade tumor staining, and 12 patients showed high grade tumor staining during the angiography (Table 3). BA was the main feeding artery for the tumor mass in all cases. The inferior phrenic artery (5 cases), esophageal artery (2 cases), internal thoracic artery (8 cases), lateral thoracic artery (2 cases), and posterior intercostal artery (4 cases) were also found as nutrient vessels of the tumor mass in some cases. The medium number of nutrient vessels was 3 (range: 1–5). Low grade SPS was detected in 15 patients and high grade SPS was found in 5 patients. The mean dose of the DEB system was 5.59 ± 2.73 ml (range: 1.5–10).

**TABLE 3:** DEB-BACE treatment.

	Tumor staining grade		
	Low	Medium	High
Total number (n)	1	7	12
Feeding artery (median, range)	2, 2	3, 2 to 5	3, 1 to 4
SPS [(Low/high) (n)]	1/0	3/4	11/1
Dose of D-BACE [M ± SD(range)]	5 ml	5.37 ± 2.35 (3–10) ml	5.6 ± 3.08 (1.5–10) ml

### Response evaluation and follow-up

**FIGURE 2:**
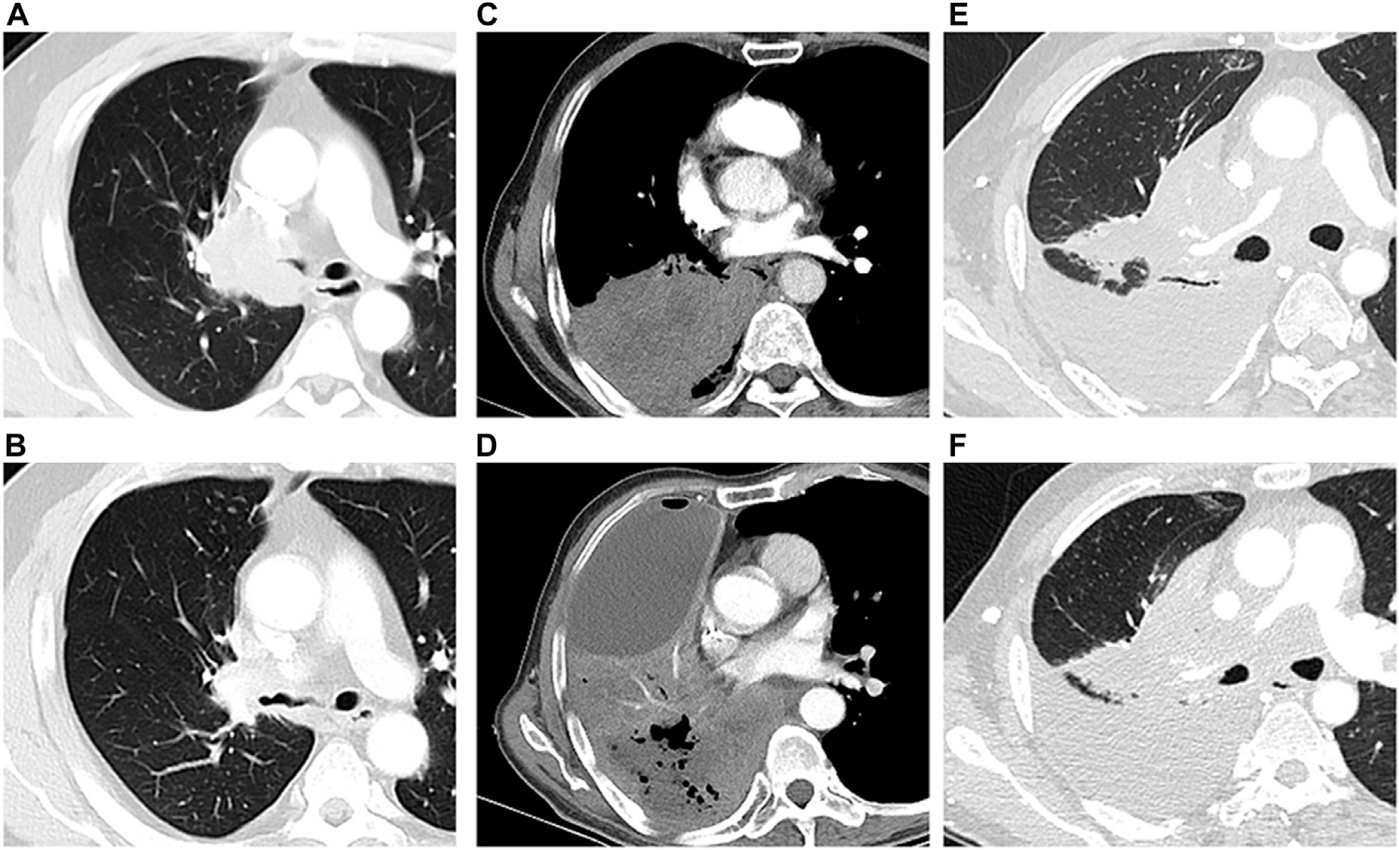
Tumor response after DEB‐BACE. One patient with solid tumor mass (squamous cell carcinoma) (**A**) achieved PR 58 days after DEB‐BACE (**B**); The tumor mass (squamous cell carcinoma) with central mild necrosis (**C**) did not respond to the DEB‐BACE, and the necrosis area became more obvious within progressive lesions (**D**); The lesion and obstructive atelectasis were stable in one patient with squamous cell carcinoma before (**E**) and after (**F**) DEB‐BACE.

Some enrolled patients failed to visit following the follow-up plan on account of the COVID-19 pandemic. Symptoms were recorded through telephone follow-up on schedule (Table 4). At the first attendance (mean time: 43.4 ± 15.26 days), CTA images revealed that 16 patients achieved PR, 1 patient achieved stable disease, and the remaining 3 patients had PD (Figure 2). ORR and DCR was 80% and 85%, respectively, and the rate of pulmonary re-expansion was 80% (16/20). The improvement rate of dyspnea was 85% and 80% at 1 week and 1 month (*p* < 0.0001, *p* < 0.0001), respectively, but relapse of dyspnea was observed in 55% of patients 2 months after DEB-BACE (*p* = 0.38). Temporary cough and phlegm aggravation was observed 1 week after DEB-BACE (*p* = 0.007, *p* = 0.002), but cough was remitted 1 month after DEB-BACE (*p* = 0.48).

**TABLE 4:** Symptoms before and after DEB-BACE.

	One week		One month		Two months	
	*Z* value	*p*-value	*Z* value	*p*-value	*Z* value	*p*-value
Dyspnea	−3.9	**<0.0001**	−3.53	**<0.0001**	−0.88	**0.38**
Cough	−2.7	**0.007**	−0.71	**0.48**	−1.86	**0.06**
Phlegm	−3.12	**0.002**	−2.13	**0.033**	−1.51	**0.13**
Fever	−3	**0.003**	−1.41	**0.157**	−1.41	**0.157**
Hemoptysis	−1	**0.317**	−1.51	**0.131**	−1.3	**0.194**
Chest pain	−2.65	**0.008**	−1.41	**0.157**	0	**1**

The bold values are the statistics values of the rank sum test, there is no special meaning.

**FIGURE 3:**
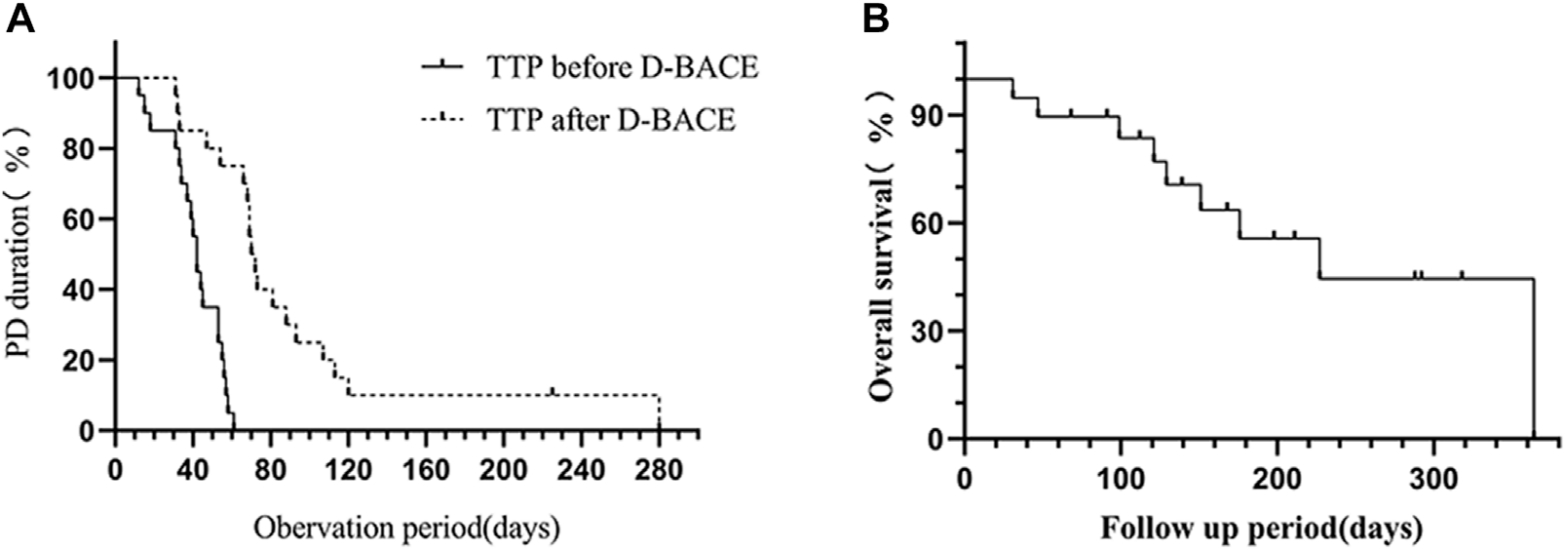
**(A)** TTP after DEB-BACE (89.55 ± 61.7 days) was significantly longer than TTP before DEB-BACE (41.25 ± 14.43 days) (*p* < 0.0001). **(B)** The OS after DEB-BACE was 238.03 ± 33.74 days (95%CI: 171.9–304.16).

TTP was 41.25 ± 14.43 days and 89.55 ± 61.7 days before and after DEB-BACE, respectively, and TTP after DEB-BACE was significantly longer compared with TTP before DEB-BACE (*p* < 0.0001) (Figure 3A). Nine patients died during the follow-up; the causes of death included tumor progression (*n* = 2), tumor cachexia (*n* = 4), respiratory failure (*n* = 2), and massive hemoptysis (*n* = 1). The OS after DEB-BACE was 238.03 ± 33.74 days (95%CI: 171.9–304.16) (Figure 3B).

The Cox regression model results indicated that tumor necrosis, longer disease duration, and longer pulmonary atelectasis duration were relevant to poor prognosis (*p* = 0.012, *p* = 0.038, *p* = 0.029). Tumor staining, feeding artery number, dose of DEB system, and SPS did not alter the effect of DEB-BACE (*p* = 0.56, *p* = 0.59, *p* = 0.15, *p* = 0.891). The remaining risk factors are listed in Table 5.

**TABLE 5:** Risk factors affecting DEB-BACE efficacy.

Risk factors	HR value	95% CI	*p*-value
Tumor staining	0.781	0.344 to 1.772	0.56
Feeding artery number	1.149	0.699 to 1.889	0.59
SPS	0.974	0.666 to 1.424	0.891
Dose of D-BACE	1.157	0.947 to 1.414	0.154
Pathology	1.047	0.486 to 2.256	0.907
Tumor stage	3.527	1.315 to 9.459	0.012
TNM Stage	1.188	0.728 to 1.939	0.49
Obstruction position	1.064	0.414 to 2.733	0.897
Degree of obstruction	0.767	0.287 to 2.052	0.597
Disease duration	1.001	1 to 1.003	0.038
Pulmonary atelectasis duration	1.01	1.001 to 1.018	0.029

### Adverse events

**FIGURE 4:**
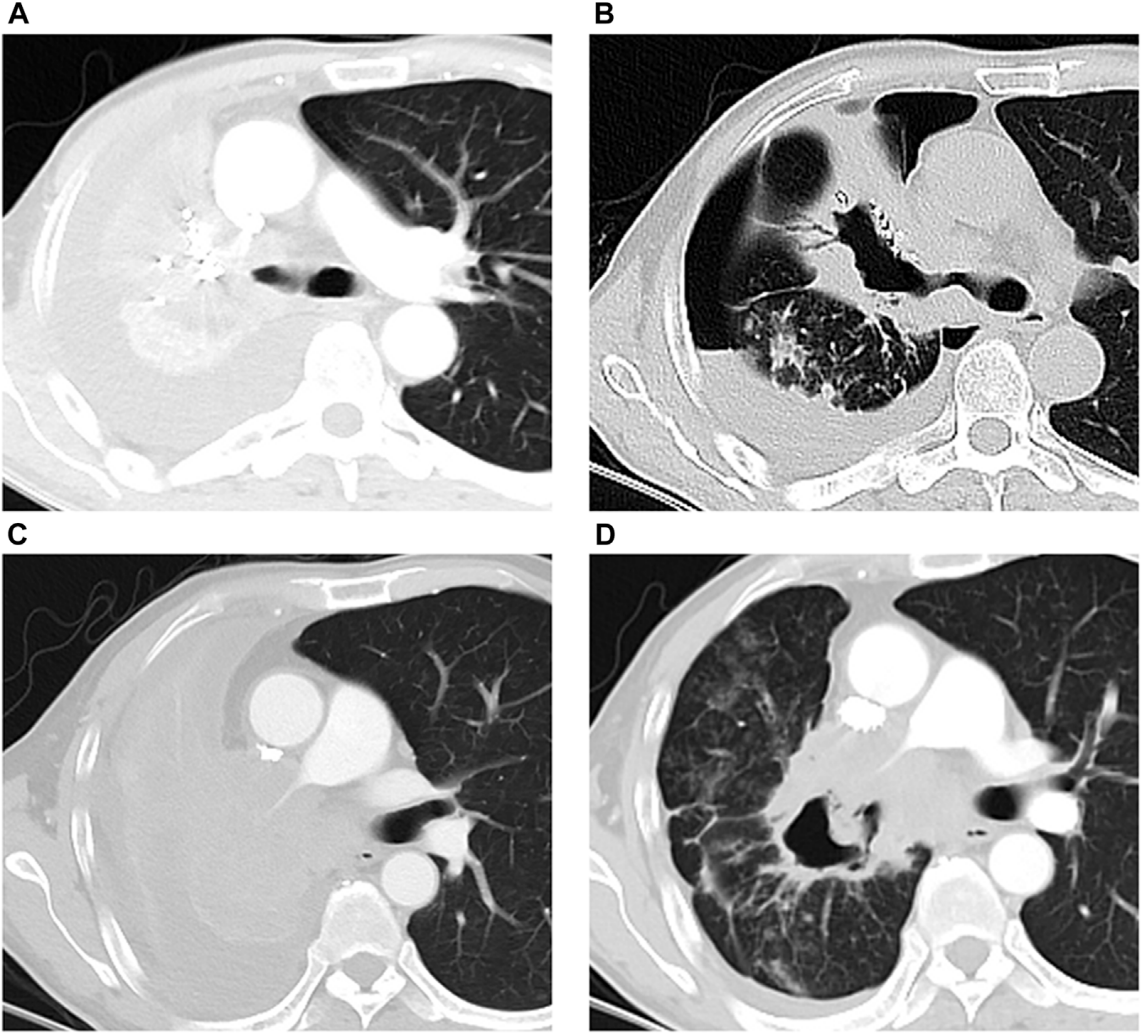
Massive hemorrhage events in two patients after DEB-BACE. **(A, B)** One patient died of massive hemoptysis related asphyxia 42 days after DEB-BACE: tumor mass with central mild necrosis **(A)** responded to DEB-BACE, and the central cavitation within tumor mass emerged **(B)** 31 days after DEB-BACE; **(C, D)** massive hemoptysis occurred 40 days after DEB-BACE: tumor mass with obstructive atelectasis **(C)** responded to DEB-BACE, the central cavitation was observed **(D)** after DEB-BACE, and the right main pulmonary artery was invaded by the tumor cavitation.

There was no occurrence of ectopic embolism during the embolization. Chemotherapy-related adverse reaction was not observed. Within 1 week after DEB-BACE, fever occurred in 9 cases (*p* = 0.003) and chest pain occurred in 12 cases (*p* = 0.008); both symptoms were relieved (*p* = 0.157, *p* = 0.157) at the 1-month follow-up (Table 3). Massive hemoptysis occurred in two cases: 1) sudden massive hemoptysis (300 ml in 1 hour) happened in one patient 40 days after DEB-BACE and he was saved by transtracheal covered-stent placement; 2) the other patient died of hemorrhage-related asphyxia at home 42 days after DEB-BACE. Moderate hemoptysis occurred in one case, and hemostasis was attempted by conservative medical therapy. In the three hemorrhage events, progressive tumor cavity was observed on CT imaging after DEB-BACE, and the main pulmonary artery (PA) was invaded by the tumor cavitation in one massive hemorrhage case basing on CTA imaging (Figure 4).

## Discussion

While multiple therapies are available and have been applied for advanced lung cancer patients, drug resistance and therapy intolerance are frequently unavoidable in the terminal stages. In progressive lung cancer, refractory obstructive atelectasis leads to a poor general condition after failure of conventional therapy. DEB-BACE is a novel therapy for advanced lung cancer, and many studies have reported the efficacy of DEB-BACE for untreated NSCLC (Liu J et al., 2021; Liu XF et al., 2021). However, few studies have described the application of DEB-BACE in lung cancer at the terminal stages (Chen. C et al., 2020; Liu X et al., 2021; Xu et al., 2022). In this study, we demonstrated the beneficial effect of DEB-BACE for progressive late-stage lung cancer after failure of IVCT, RT, MTT and PD-1. This treatment could control disease progression and contribute to pulmonary re-expansion over a relatively long period of time (Shang et al., 2020). Improvement of dyspnea would elevate patient quality of life and strengthen confidence for patients with advanced lung cancer after failure of various therapies (Zhao et al., 2020). Comparing the high pulmonary re-expansion rate after airway stent placement as the previous study reported, DEB-BACE would achieve effective pulmonary re-expansion, and meanwhile still locally control tumor growth (Bi et al., 2020).

The CalliSpheres bead is capable of loading a variety of chemotherapy drugs, including gemcitabine and vinorelbine, which are used to treat lung cancer. The previous studies have reported the good efficacy of pirarubicin or gemcitabine-loaded drug-eluting beads combined with BAI (Li et al., 2021; Liu J et al., 2021; Liu X et al., 2021; Bi et al., 2022). In our study, all enrolled patients had experienced IVCT resistance (gemcitabine, docetaxel, and cis-platinum or carboplatin), and therefore vinorelbine was optional as the second-line chemotherapy drug. Different from the previous study, the dose of chemotherapy drugs had to be reduced on account of patients having a poor general condition in our study. This requires CalliSpheres beads to have a high affinity for a low dose of chemotherapy drugs to maintain an effective concentration. A previous study demonstrated the high entrapment efficiency (up to 90%), fast loading speed (attaining 90% loading rate within 10 min), and high release rate (attaining 100% releasing rate within 60 min) of vinorelbine-load beads (Wang et al., 2020). Hence, vinorelbine was a suitable option in this study. In our study, a low dose of vinorelbine within the tumor mass exerted efficient anti-tumor effects, while having minimal side effects (Lewis and Dreher, 2012; Fu et al., 2016; Bie, Li, Li, Wang, Li, et al., 2019).

CalliSpheres beads have a smooth surface and excellent compressive elasticity (up to 50%). Additionally, the size of beads can be reduced by 40% after drug loading. These characteristics seem to increase the risk of ectopic embolism, especially for a tumor mass accompanied by SPS (Gadaleta et al., 2013). The previous study has also indicated that embolic beads smaller than 200 µm could lead to pulmonary infarction and ectopic embolism. In consideration of compressive elasticity and shrinkage of beads after drug loading, the CalliSpheres beads smaller than 300 µm was potentially dangerous during embolization (Gadaleta et al., 2013). We did not observe ectopic embolism in the study group, and beads with a diameter of 300–500 µm could achieve satisfying effects in the embolization of tumor nutrient vessels (S. Zhang et al., 2017). The drug-loaded beads were able to achieve an effective anchoring effect within the tumor microvasculature (Mikhail et al., 2018). The drug is slowly released from the DEB system into the surrounding tumor and achieves a relatively high concentration in a short time, which kills the tumor, while the beads cut off the nutrient blood flow (Namur et al., 2011; Dreher et al., 2012). The DEB system is a safe and effective treatment for controlling tumor growth.

In our study, we found that tumor necrosis was relevant to the poor efficacy of DEB-BACE. A poor blood supply within tumors with necrosis would not ensure anchoring of the DEB system within the tumor microvasculature. Long disease duration and long pulmonary atelectasis duration were related to poor prognosis, and it reminded us that prompt DEB-BACE could benefit patients by achieving timely pulmonary re-expansion (Shang et al., 2020). While abundant blood supply to tumors predicted good prognosis of BAI in previous studies (Nakanishi et al., 2012), we observed that a solid tumor mass with a poor blood supply also showed a good response after DEB-BACE. We believe that anchoring DEB systems within the tumor microvasculature may slowly release vinorelbine to maintain higher effective anti-tumor drug concentrations than BAI. Additionally, embolization of DEB systems may also reduce drug loss on account of the presence of SPS.

Hemoptysis after DEB-BACE should be especially noted. In our study, three hemorrhage AEs occurred in patients who achieved PR and showed tumor cavitation. The exposure of the vascular structure caused by the emerging tumor cavitation erosion is likely the main reason, and in particular the impaired wall of the central pulmonary artery will result in life-threatening hemorrhage in a very short time (Dansin et al., 2012; Anscher et al., 2022). In this dangerous hemorrhage situation, BA embolization and BACE will be ineffective, and embolization or covered-stent placement of PA and covered airway stent placement will be better options (Lee et al., 2012; Marcelin et al., 2018; Gershman et al., 2019). During the follow-up, the emerging tumor cavitation should be properly identified, and CTA is a useful method to monitor cavitation (Khalil et al., 2008; Reck et al., 2012).

This study has some limitations. This was a prospective single-arm study, and thus comparison of the advantage of DEB-BACE with other therapies requires further investigation. Furthermore, this study is limited by a small sample size, and the safety and efficacy of DEB-BACE requires analysis in multi-center and large-sample clinical studies.

In conclusion, DEB-BACE is a feasible and safe treatment for advanced lung cancer with progressive obstructive atelectasis, and it is conductive to the local control of tumor after failure of conventional therapies. Prompt treatment is recommended for improving the quality of life and strengthening the confidence of patients, and the combination therapy of DEB-BACE and other treatments is worthwhile to apply to the advanced lung cancer in the future.

## Data Availability

The raw data supporting the conclusions of this article will be made available by the authors, without undue reservation.
